# Vascular RAGE transports oxytocin into the brain to elicit its maternal bonding behaviour in mice

**DOI:** 10.1038/s42003-019-0325-6

**Published:** 2019-02-25

**Authors:** Yasuhiko Yamamoto, Mingkun Liang, Seiichi Munesue, Kisaburo Deguchi, Ai Harashima, Kazumi Furuhara, Teruko Yuhi, Jing Zhong, Shirin Akther, Hisanori Goto, Yuya Eguchi, Yasuko Kitao, Osamu Hori, Yoshitake Shiraishi, Noriyuki Ozaki, Yu Shimizu, Tomoya Kamide, Akifumi Yoshikawa, Yasuhiko Hayashi, Mitsutoshi Nakada, Olga Lopatina, Maria Gerasimenko, Yulia Komleva, Natalia Malinovskaya, Alla B. Salmina, Masahide Asano, Katsuhiko Nishimori, Steven E. Shoelson, Hiroshi Yamamoto, Haruhiro Higashida

**Affiliations:** 1 Department of Biochemistry and Molecular Vascular Biology, Kanazawa University Graduate School of Medical Sciences, Kanazawa, 920-8640 Japan; 2 Department of Basic Research on Social Recognition and Memory, Research Centre for Child Mental Development, Kanazawa University, Kanazawa, 920-8640 Japan; 3 Medical Research Institute, Kanazawa Medical University and Medical Care Proteomics Biotechnology Co., Uchinada, Ishikawa, 920-0293 Japan; 4 Department of Neuroanatomy, Kanazawa University Graduate School of Medical Sciences, Kanazawa, 920-8640 Japan; 5 Department of Functional Anatomy, Kanazawa University Graduate School of Medical Sciences, Kanazawa, 920-8640 Japan; 6 Department of Neurosurgery, Kanazawa University Graduate School of Medical Sciences, Kanazawa, 920-8640 Japan; 7 Laboratory for Social Brain Studies, Research Institute of Molecular Medicine and Pathobiochemistry, and Department of Biochemistry, Krasnoyarsk State Medical University, Krasnoyarsk, Russia 660022; 8 Division of Transgenic Animal Science, Kanazawa University Advanced Science Research Centre, Kanazawa, 920-8640 Japan; 9 Laboratory of Molecular Biology, Department of Molecular and Cell Biology, Graduate School of Agricultural Science, Tohoku University, Sendai, 981-8555 Japan; 10 Joslin Diabetes Centre & Harvard Medical School, Boston, MA 02215 USA; 11 Komatsu University, Komatsu, 923-0921 Japan

## Abstract

Oxytocin sets the stage for childbirth by initiating uterine contractions, lactation and maternal bonding behaviours. Mice lacking secreted oxcytocin (*Oxt*^−/−^, *Cd38*^−/−^) or its receptor (*Oxtr*^−/−^) fail to nurture. Normal maternal behaviour is restored by peripheral oxcytocin replacement in *Oxt*^−/−^ and *Cd38*^−/−^, but not *Oxtr*^−/−^ mice, implying that circulating oxcytocin crosses the blood-brain barrier. Exogenous oxcytocin also has behavioural effects in humans. However, circulating polypeptides are typically excluded from the brain. We show that oxcytocin is transported into the brain by receptor for advanced glycation end-products (RAGE) on brain capillary endothelial cells. The increases in oxcytocin in the brain which follow exogenous administration are lost in *Ager*^−/−^ male mice lacking RAGE, and behaviours characteristic to abnormalities in oxcytocin signalling are recapitulated in *Ager*^−/−^ mice, including deficits in maternal bonding and hyperactivity. Our findings show that RAGE-mediated transport is critical to the behavioural actions of oxcytocin associated with parenting and social bonding.

## Abstract (web summary)

Yasuhiko Yamamoto et al. show that oxytocin is transported into the brain by the receptor for advanced glycation end-products (RAGE) on the blood-brain barrier. This study explains how circulating oxytocin crosses the blood-brain barrier, which is important to manifest oxytocin’s maternal bonding effects.

## Introduction

Oxytocin mediates both physiological and psychosocial events surrounding mammalian birth, including uterine contractions, initiation of lactation, and maternal bonding^1,2^. Oxytocin produced by oxytocinergic neurons in hypothalamic paraventricular and supraoptic nuclei (PVN, SON) has both central and peripheral actions, the latter through posterior pituitary release into the circulation^1–6^. Systemic loss of either oxytocin or oxytocin receptors (*Oxt*^−/−^ and *Oxtr*^−/−^) disrupts maternal nurturing behaviours in mice^7,8^. This is restored by administration of exogenous oxytocin in *Oxt*^−/−^ but not *Oxtr*^−/−^ mice, suggesting that peripheral oxytocin has central effects. Mice lacking CD38, a cyclic ADP ribose synthetase and hydrolase necessary for secretion of oxytocin, also display this characteristic behavioural phenotype, which is also reversed following administration of exogenous oxytocin^5,9^. Exogenous oxytocin in humans also appears to have behavioural effects, particularly in such social deficit-related psychiatric disorders as autism and schizophrenia^9–13^. Numerous clinical trials are determining the psychopharmacological effects of peripherally administered oxytocin in a variety of conditions^14,15^. The potential for peripherally administered oxytocin to act centrally is supported by measures of it in the brain. Oxytocin concentrations are maximal in human or primate cerebrospinal fluid (CSF) and mammalian hippocampus and amygdala within 10–60 min of peripheral administration^16–19^. Because many peripheral peptides and proteins do not pass freely into the central nervous system (CNS)^20,21^, we hypothesised a specialised uptake mechanism for oxytocin to cross the blood-brain barrier (BBB) and gain access to the CNS.

RAGE, a member of the immunoglobulin superfamily of pattern recognition receptors, is not known to be related to oxytocin or oxytocin signalling^22–33^. Full-length, membrane bound RAGE (mRAGE) is present on many cell types. Endogenous soluble RAGE (esRAGE), the product of an alternatively spliced mRNA, is found in the circulation as it lacks a membrane-spanning domain^29,34^. A second soluble form, ectodomain-shed RAGE (sRAGE), is a proteolytic product of mRAGE^28,30^. The three RAGE forms bind various ligands with similar affinities, including multiple advanced glycation end-products (AGEs) and amyloid β peptide^31–37^.

Here, our findings show that the expression of RAGE on capillary endothelial cells of the blood-brain barrier (BBB) is both necessary and sufficient for the transport of oxytocin into the brain. The increases in oxytocin in the brain which follow exogenous administration are lost in RAGE knockout male mice (*Ager*^−/−^ mice lacking the mouse *receptor for AGEs* gene). Behaviours characteristic to abnormalities in oxytocin signalling are recapitulated in *Ager*^−/−^ mice, including deficits in maternal bonding and hyperactivity, and both transport and behavioural deficits are restored in *Ager*^−/−^ mice following transgenic, endothelial cell expression of RAGE. These findings indicate that RAGE-mediated transport can explain the degree of brain oxytocin recruitment previously reported at the molecular level, and demonstrate that oxytocin is critical to the behavioural actions of oxytocin associated with parenting and social bonding.

## Results

### Oxytocin-RAGE interactions

**Fig. 1:**
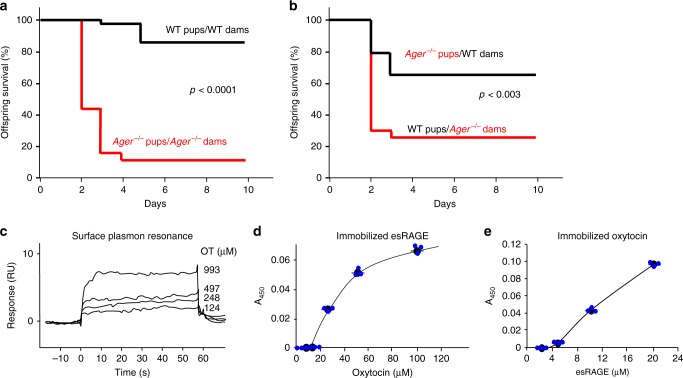
Offspring survival and RAGE-oxytocin interactions. Kaplan–Meier survival curves for offspring of WT (*Ager*^+/+^) and RAGE-null *Ager*^−/−^ (knockout of the mouse gene for the receptor for AGEs) dams housed with their biological mothers (**a**; *n* = 40 WT pups/WT dams; 38 *Ager*^−/−^pups/*Ager*^−/−^ dams), or transferred to different cages and housed with postpartum dams of the opposite genotype (**b**; *n* = 66 WT pups/*Ager*^−/−^ dams; 66 *Ager*^−/−^ pups/WT dams). *P-*values derived from log-rank calculations. **c** Surface plasmon resonance (BiaCore) assessment of oxytocin binding to immobilised human esRAGE. **d**, **e** Microtitre plate wells contain varying amounts of immobilised oxytocin (**d**) or esRAGE (**e**); added **d** esRAGE (1 μg/ml) or **e** oxytocin (100 μM) were detected immunochemically (*n* = 3). Values are mean ± SEM

Survival of *Ager* gene null (*Ager*^−/−^) pups is often low due to maternal neglect, with even mild maternal stress leading to dramatic reductions in offspring survival (Fig. 1a). This is clearly a maternal deficit, as newborn *Ager*^−/−^ pups transferred to wild-type (WT) post-partum mothers are nurtured and survive at near normal frequencies. By contrast, normal offspring of WT mothers do poorly when transferred to post-partum *Ager*^−/−^ mothers (Fig. 1b). Offspring neglect in the absence of gross anatomic or histological defects is a behavioural deficit reminiscent of the characteristic disorders seen with loss of oxytocin function, as in *Oxt*^−/−^, *Oxtr*^−/−^ or *Cd38*^*−*/−^ mice^5,7–9,38–40^. Survival rates for *Ager*^−/−^ pups fostered by WT mothers were lower than for WT offspring raised by their WT mothers, and survival rates for WT pups transferred to *Ager*^−/−^ mothers tend to be higher than for *Ager*^−/−^ offspring raised by their *Ager*^−/−^ mothers (Fig. 1a, b). This too is seen in *Oxt*^−/−^, *Oxtr*^−/−^, or *Cd38*^*−*/−^ mice and attributable to intrauterine and peripartum effects of loss of oxytocin function occurring prior to the exchange of offspring. We therefore hypothesised potential interactions between oxytocin and RAGE signalling pathways.

Multiple methods were used to demonstrate direct binding of oxytocin and RAGE. Surface plasmon resonance methods showed concentration-dependent oxytocin binding to immobilised recombinant esRAGE, with an apparent dissociation constant (*K*_D_) of 179 nM (Fig. 1c). Oxytocin-RAGE binding was further confirmed using plate assays where either oxytocin or esRAGE was immobilised, and esRAGE or oxytocin was in solution, respectively (Fig. 1d, e).

Endogenous oxytocin and soluble RAGE also associate in human circulation. Using gel permeation chromatography to separate serum proteins based on molecular size, oxytocin immunoreactivity co-eluted with soluble RAGE (Supplementary Figure 1a). Larger amounts of free oxytocin eluted later, as the molar amount of oxytocin in blood exceeds that of soluble RAGE. Endogenous oxytocin in human serum (30 pmol/50 ml) also co-eluted with soluble RAGE following isolation by anti-RAGE antibody affinity chromatography (Supplementary Figure 1b). Mass spectrometry was employed for further structural identification. In this case, we used immobilised esRAGE to isolate oxytocin from 50 ml human serum, and repeated the procedure after adding a synthetic oxytocin standard (100 ng) (Supplementary Figure 1c). Analyses of the eluted fractions by liquid chromatography-tandem mass spectrometry (LC-MS/MS) showed that endogenous oxytocin in human serum is structurally indistinguishable from the synthetic oxytocin standard, as they eluted at identical times (Supplementary Figure 1d) and have identical parent ions (M+H^+^ 1008.2). Mass spectrometry of the eluted material from the immobilised esRAGE did not identify structurally related peptides such as arginine-vasopressin which may cross-react in immunoassays.

Oxytocin binding to soluble RAGE suggests it also binds membrane RAGE, but oxytocin neither stimulates a RAGE-dependent NF-κB reporter nor inhibits reporter induction by AGEs, a ligand that activates the NF-κB reporter^22,27^ (Supplementary Figure 2a). Furthermore, oxytocin did not activate NF-κB-independent Rac1 or Cdc42 signalling, which are also suggested to function downstream of RAGE^24,41,42^ (Supplementary Figures 2b and 2c). Therefore, while oxytocin binds soluble and presumably membrane RAGE, it neither induces nor inhibits intracellular signals reported for RAGE ligands. Moreover, oxytocin binding to soluble RAGE was weakly or non-specifically inhibited by potential RAGE ligands: S100B, AGEs, amyloid β or high-mobility group B1 (HMGB1)^24,35,42^ (Supplementary Figures 3a–d).

To further explore potential oxytocin-RAGE binding modes, we subdivided extracellular RAGE into its V, C1 and C2 domains. S100B, AGEs, amyloid β, and HMGB1 all bind the RAGE V domain^24,28^. Oxytocin binding to soluble RAGE was blocked by addition of recombinant V domain, marginally inhibited by C1 domain, and not blocked by C2 domain (Supplementary Figure 3e), suggesting that oxytocin binds the V domain, but at a distinct site from S100B, AGEs, amyloid β or HMGB1^42,43^.

### Oxytocin transport across the BBB

**Fig. 2:**
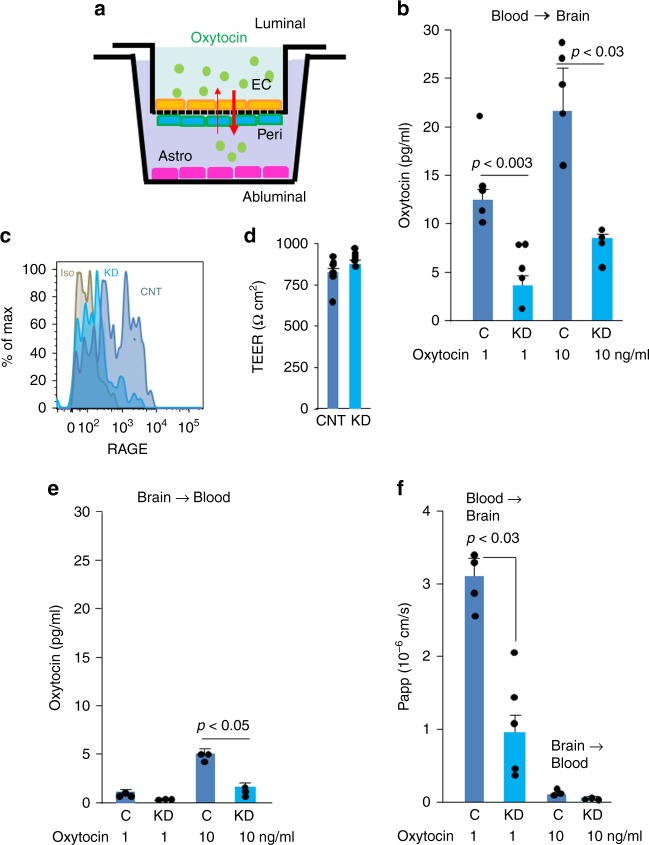
RAGE transports oxytocin across an in vitro blood brain barrier (BBB). **a** A schematic diagram of the monkey BBB kit (PharmaCo-Cell). EC, monkey brain capillary endothelial cells; Peri, rat brain pericytes; Astro, rat astrocytes. The upper and the lower chambers represent the luminal (blood) and abluminal (brain extracellular space) sides, respectively. **b** Oxytocin (1 or 10 ng/ml) was added to the upper (luminal, blood) chambers of the model BBB system, and 3 h later oxytocin was quantified in the lower (abluminal, brain) chambers (*n* = 4–5). **c** Flow cytometry. The endothelial cells used in the upper chamber in **a** were treated with RAGE shRNA (knockdown, KD) or control (C or CNT) vectors to assess the effects of RAGE knockdown. Isotype control (Iso) instead of anti-RAGE antibody assesses background signal. **d** Transendothelial electrical resistance (TEER, Ωcm^2^) measures >150 Ωcm^2^ indicate integrity of the model BBB (*n* = 6). **e** Conversely, oxytocin was added to the abluminal chambers and its transport to the luminal chamber was quantified (*n* = 3). **f** The apparent permeability constants (Papp) for transfer were calculated from the distribution ratios across the chambers (*n* = 3–5). Values are mean ± SEM

In vitro model systems are used to predict the ability of compounds to cross into the CNS and exert central effects^44,45^. We used one such system constructed using primary cultures of monkey brain capillary endothelial cells (EC) coupled with rat pericytes and astrocytes to assess requirements^46^ for endothelial RAGE in the transport of oxytocin (Fig. 2a). Endothelial transport of oxytocin was dose-dependent (Fig. 2b) and of a similar rate to transport of centrally acting drugs^46^. RAGE was either left at endogenous levels or reduced in the ECs by shRNA knockdown (Fig. 2c). The integrity of the in vitro primate “BBB” was unaffected by RAGE knockdown, assessed by high transendothelial electrical resistance (TEER) (Fig. 2d). Notably, oxytocin transport was reduced selectively in the blood-to-brain direction following RAGE knockdown (Fig. 2b). Reverse transport was much less efficient (Fig. 2e), and according to calculated apparent permeability (Papp) measures, inconsequential (Fig. 2f).

**Fig. 3:**
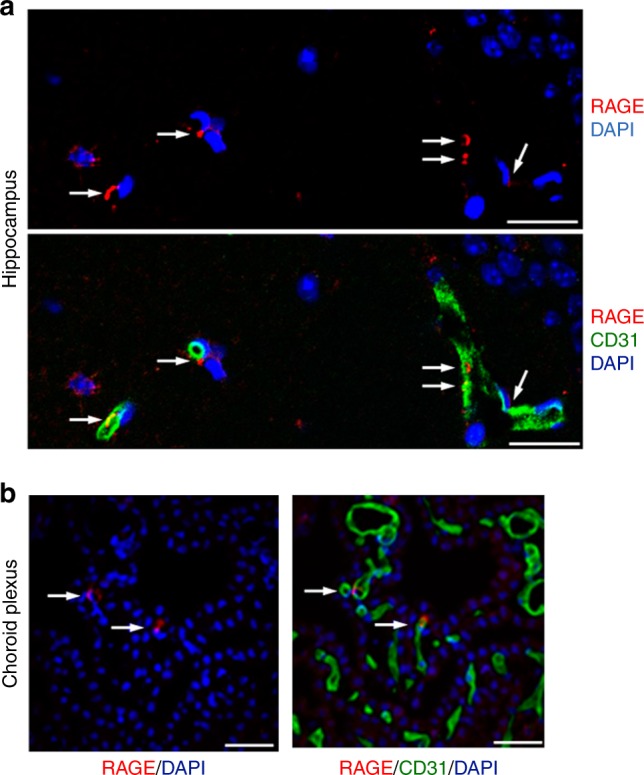
RAGE in the brain vasculature. **a**, **b** Confocal microscopy. Sections of the hippocampus (**a**; CA1, stratum radiatum) and choroid plexus in the third ventricle (**b**) of WT male mice were immunostained with anti-RAGE and anti-CD31 antibodies; nuclei were stained with DAPI (Bar = 100 μm). Co-staining of RAGE (red, arrows) with CD31 (green) indicates that RAGE is present in some vascular endothelial cells

Immunohistochemical analyses showed RAGE in vascular patterns limited to CD31 (platelet endothelial cell adhesion molecule)-positive endothelial cells^23^, which are the components of the BBB, of the hippocampus and choroid plexus of WT (Fig. 3a, b) but not of *Ager*^*−/−*^ mice (Supplementary Figure 4a). We also performed confocal microscopic analyses to observe RAGE expression in the circumventricular organs (CVOs) of the brain, referred to as ‘windows of the brain’, and found that RAGE expression was clearly observable in the endothelial cells of the neurohypophysis (Supplementary Figure 4b), but barely in those of the sensory CVOs, including the organum vasculosum of the lamina terminalis, subfornical organ, and area postrema. Further analysis revealed co-localisation of RAGE with cavelolin-1, a marker of plasma membrane invaginations (caveolae), in endothelial cells and the choroid plexus in WT mice (Supplementary Figure 4c), suggesting a role of RAGE in the oxytocin transfer (trafficking) through caveolae-associated endocytosis and transcytosis (Supplementary Figure 4d). By contrast, oxytocin receptors, which could theoretically act as oxytocin transporters, were not co-localised with endothelial cells in numerous sections of 5 brain regions of Venus mice expressing a fluorescent oxytocin receptor reporter^47^ (Supplementary Figures 4e and f).

### RAGE-dependent transport of oxytocin across the BBB

**Fig. 4:**
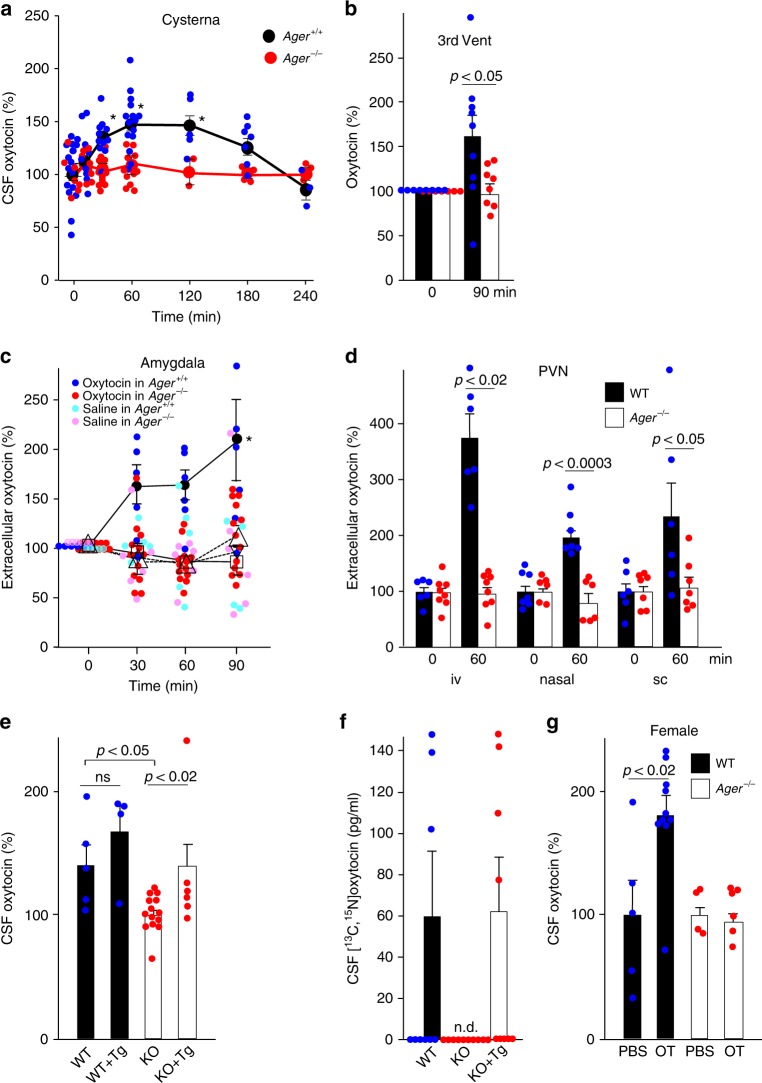
Transport of oxytocin into the brain. **a** Oxytocin concentrations in CSF from the cisterna magna after subcutaneous injection of 30 ng oxytocin in WT (*Ager*^+/+^) or *Ager*^−/−^ male mice (*n* = 3–15/data point, *p < 0.05). **b** Oxytocin concentrations in the CSF of the third ventricles before and 90 min after subcutaneous administration of oxytocin (30 ng) (*n* = 7–9). **c** Oxytocin concentrations in microperfusates of the amygdala before and after intranasal (20 ng) oxytocin. Closed circles, oxytocin in WT mice; open triangles, oxytocin in *Ager*^−/−^ mice; inverted open triangles, saline in WT mice; open squares, saline in *Ager*^−/−^ mice (*n* = 4–13; *p < 0.05). **d** Oxytocin concentrations in microperfusates of the paraventricular nuclei (PVN) of WT mice before and 60 min after intravenous (iv), nasal (in) or subcutaneous (sc) administration of oxytocin (*n* = 6–8). **e**, **f** Transgenic (Tg) mice expressed human RAGE selectively in endothelial cells either on WT or *Ager*^−/−^ (KO) backgrounds. Oxytocin concentrations in the CSF were measured 60 min after subcutaneous injections of oxytocin (**e**; *n* = 4–14) or [^13^C,^15^N]oxytocin (**f**; *n* = 9–10). **g** Oxytocin concentrations in the CSF of female mice were measured 60 min after subcutaneous injections of oxytocin (OT) (*n* = 4–10). n.d., not detected; ns, not significant. Values are mean ± SEM

Oxytocin concentrations in blood and CSF were assessed following a subcutaneous injection of oxytocin (30 ng/mouse; approximately 1.1 μg/kg of body weight). Steady-state levels in blood (~20 pg/ml) increased rapidly, peaking within 10 min after injection and returning to baseline by 1–2 h in both WT and *Ager*^−/−^ male mice (Supplementary Figure 5a). By contrast, levels of oxytocin in CSF from the cisterna magna of WT mice increased more gradually to maxima at 1–2 h before returning to baseline at 4 h (Fig. 4a). Despite equivalently high serum levels following injection of identical amounts of oxytocin (Supplementary Figure 5a), concentrations in the CSF of *Ager*^−/−^ mice remained at baseline levels (Fig. 4a).

In separate experiments we cannulated the third ventricles of WT and *Ager*^−/−^ mice and withdrew CSF for oxytocin measurements. Following subcutaneous injection, oxytocin increased in third ventricle CSF of WT but not *Ager*^−/−^ male mice (Fig. 4b and Supplementary Figure 5b). Microperfusion methods showed that oxytocin also increased in relevant regions of the brain, including the amygdala and PVN of the hypothalamus, of WT but not *Ager*^−/−^ male mice following subcutaneous, intravenous or intranasal administration of oxytocin but not saline carrier (Fig. 4c, d and Supplementary Figure 5c). The direct CSF measures (Fig. 4a, b) and microperfusion results (Fig. 4c, d) confirm that oxytocin in the blood is transported across the BBB as suggested by Landgraf and his collegues^16,17,20^ and to our knowledge, show for the first time that endothelial RAGE is required for oxytocin transport into the CNS.

Because this is such an important point, we further investigated oxytocin transport following selective transgenic (Tg) expression of RAGE in endothelial cells of both WT and *Ager*^−/−^ mice using an *Flk-1* promoter^26,41^. Protein expression in both lines was equivalent to the amount of endogenous endothelial RAGE in WT mice (WT = 1, WT + Tg~2, KO + Tg~1). CSF concentrations were measured following subcutaneous injections of oxytocin. Tg expression in *Ager*^−/−^ mice increased oxytocin in CSF to the WT concentration, whereas Tg expression in WT mice minimally affected CSF oxytocin concentrations (Fig. 4e and Supplementary Figure 5d). Stable isotope-labelled oxytocin was used to more accurately assess transport into the CSF. Following subcutaneous injection, [^13^C,^15^N]oxytocin was undetectable in CSF of *Ager*^−/−^ male mice, whereas amounts in the CSF of WT or KO + Tg mice were equivalent (Fig. 4f and Supplementary Figure 5e). Although oxytocin has been found to produce sex-specific effects^48–50^, RAGE-mediated oxytocin transport was also observed in female WT-mice (Fig. 4g). These results demonstrated that RAGE is both necessary and sufficient for oxytocin transport into the brain in both male and female mice.

**Fig. 5:**
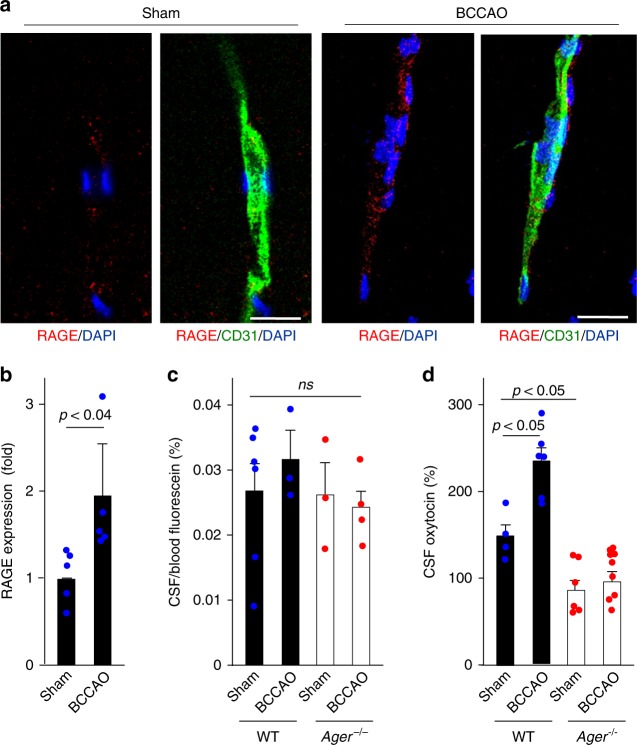
Oxytocin transport after transient brain ischaemia. **a** Transient brain ischaemia was induced by 15 min of bilateral common carotid arteries occlusion (BCCAO) as described in Methods. RAGE and CD31 expression in vascular cells of the hippocampus were assessed 24 h after ischaemic insults; nuclei are stained with DAPI (Bar = 100 μm). **b** Quantitation of RAGE induction in CD31-positive endothelial cells (*n* = 5). **c** Fluorescein dye was used to check nonspecific vascular leakage and BBB damage following BCCAO (*n* = 3–6). ns, not significant. **d** Oxytocin concentrations in CSF from the cisterna magna of BCCAO or sham-operated WT (RAGE^+/+^) or *Ager*^−/−^ male mice (*n* = 4–9). Values are mean ± SEM

Endothelial RAGE is upregulated in brain capillaries after bilateral common carotid artery occlusion (BCCAO)^23^. Using this method we found a ~2-fold increase in capillary RAGE (Fig. 5a, b). We predicted this would also promote oxytocin transport. As a control, we showed this procedure does not cause fluorescein to leak into the CSF (Fig. 5c), suggesting there is not a generalised increase in vascular permeability. Nevertheless, exogenous oxytocin increased in the CSF of WT but not *Ager*^−/−^ male mice after bilateral carotid occlusion (Fig. 5d and Supplementary Figure 5f).

### c-Fos as CNS actions of oxytocin

**Fig. 6:**
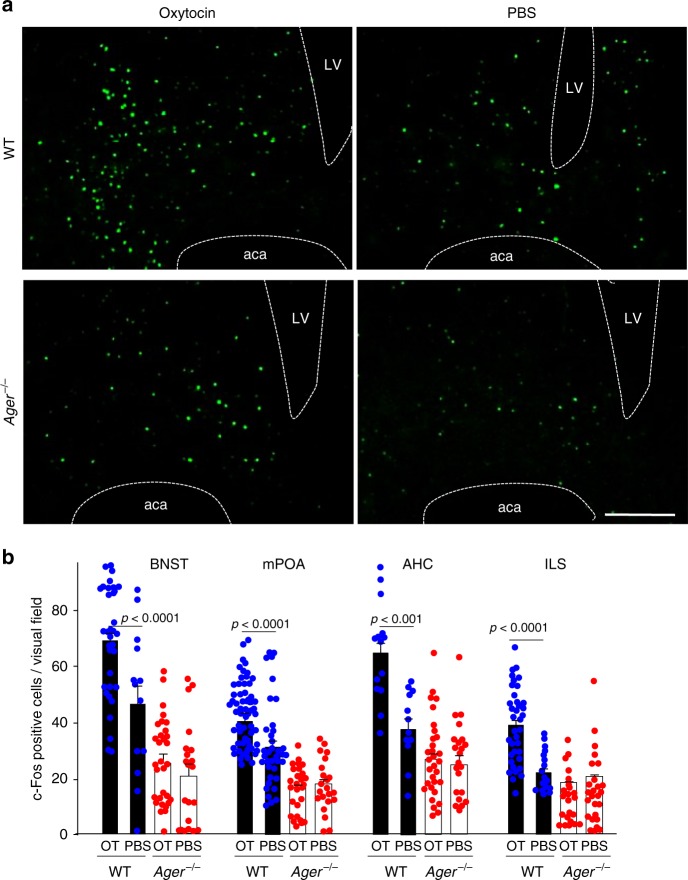
Reporter assay for oxytocin activity in the brain. **a** 60 min after sc administration of oxytocin (OT) (30 ng) or PBS, c-Fos positive nuclei were visualised in the bed nucleus of the stria terminalis (BNST) of WT and *Ager*^−/−^ male mice (LV, lateral ventricle; aca, anterior commissure, anterior part) (Bar = 100 μm). **b** Densities of c-Fos positive nuclei in the BNST, medial preoptic area (mPOA), centre of the anterior hypothalamic area (AHC), and intermediate lateral septal nucleus (ILS) regions of WT and *Ager*^−/−^ mice (*n* = 12–56)

The classical studies of Numan showed c-Fos positive neurons are activated during the acquisition of postpartum maternal behaviours^51^. We utilised the nuclear translocation of c-Fos as a readout for the neuronal actions of oxytocin^52^, which are now known to activate c-Fos positive neurons^51–53^. Numbers of c-Fos positive nuclei increased after subcutaneous oxytocin administration in four distinct regions of the brain, the bed nucleus of the stria terminalis (BNST), medial preoptic area (mPOA), centre of the anterior hypothalamic area (AHC) and ventral portion of the intermediate lateral septal nucleus (ILS) (Fig. 6a, b). This is dependent on both oxytocin and RAGE, as there are no effects in *Ager*^−/−^ male mice or when vehicle control was administered (Fig. 6b).

### Maternal behaviour

**Fig. 7:**
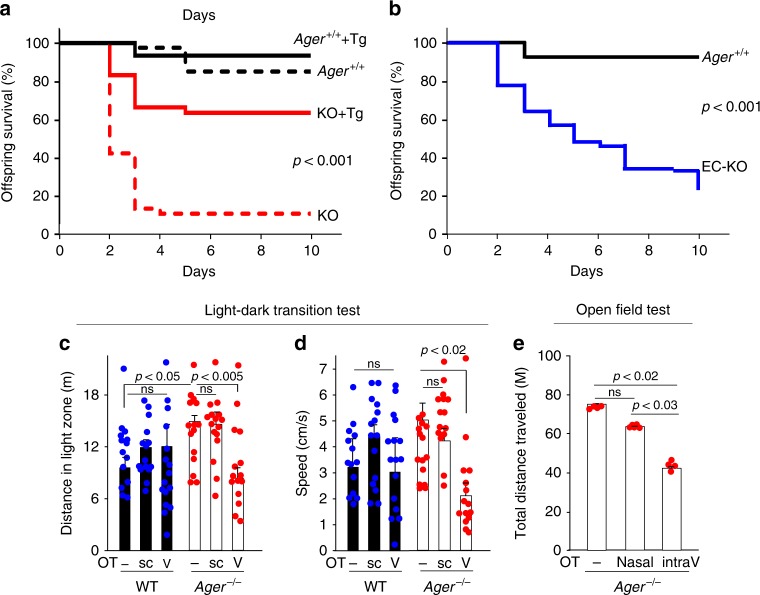
Offspring survival in *Ager*^−/−^ mice and behavioural characteristic. **a** Survival curves for biological offspring of WT (*Ager*^+/+^) dams with (solid black line, *n* = 45) or without (black dashed line, *n* = 40) re-expressed human RAGE (Tg). Survival curves for biological offspring of *Ager*^−/−^ (KO) dams with (red solid line, *n* = 71) or without (red dashed line, *n* = 38) re-expression of human RAGE. *P* < 0.001. **b** Survival curves for offspring of dams deficient for RAGE in endothelial cells (EC-KO, *n* = 76) and WT (*Ager*^+/+^) dams (solid black line, *n* = 45). *P* < 0.001. *P*-values derived from log-rank calculations. **c**, **d** During light-dark transition tests, the greater distances travelled (**c**) and greater average speed (**d**) in the light zone exhibited by *Ager*^−/−^ male mice were both reduced by intraventricular (V) but not subcutaneous (sc) injection of oxytocin (OT) (*n* = 14–16). **e** In open field tests that assess anxiety in a new environment, the total distance travelled during the first 5 min was determined for *Ager*^−/−^ male mice before and after intraventricular (intraV) (5 min, 0.1 ng/μl × 3 μl/min) or nasal (100 ng/ml × 20 μl) administration of oxytocin (OT) (*n* = 4–5). ns, not significant. Values are mean ± SEM

Loss of maternal nurturing and social interactive behaviours in *Cd38*^−/−^ mice have been restored following peripheral administration of oxytocin, consistent with transport of peripherally administered exogenous oxytocin into brain^5^. We would not expect this to reverse the maternal bonding deficit in RAGE-null *Ager*^−/−^ mice, as endothelial RAGE appears to be required for CNS entry of peripheral oxytocin. Survival of *Ager*^−/−^ pups is often low due to maternal neglect, with even mild maternal stress leading to dramatic reductions in offspring survival (Fig. 7a). However, our findings predict that restoration of endothelial RAGE in *Ager*^−/−^ mice would reverse the behavioural phenotypes. There was a substantial rescue of the *Ager*^−/−^ phenotype, as 63.4% of the transgenic *Ager*^−/−^ (KO + Tg) offspring survived, compared to only 10.5% survival for the non-transgenic *Ager*^−/−^ (KO) offspring (*P* *<* 0.001) (Fig. 7a). Litters contained both transgenic *Ager*^−/−^ and non-transgenic *Ager*^−/−^ offspring, and all offspring were raised by their respective birth mothers. In addition, offspring survival rate was lower in endothelial RAGE-deficient (EC-KO) mothers (*P* *<* 0.001; Fig. 7b).

### Social behaviour in RAGE knockout mice

Clinical studies have linked autism and Asperger’s disorders and attention-deficit/hyperactivity disorders to oxytocin deficiency^7,8,54^, and the potential use of exogenous oxytocin to treat these conditions is being assessed in clinical trials^10,11,54–57^. *Ager*^−/−^ male mice are also known to be hyperactive, with greater speeds and distances travelled during both light-dark transition and open field tests^58^. These have been suggested to be potential mouse equivalents of human anxiety properties. We therefore reasoned that the hyperactivity seen in *Ager*^−/−^ male mice might also be related to a deficiency in transport of peripheral oxytocin into the CNS, and administered exogenous oxytocin to WT and *Ager*^−/−^ male mice. Normal activity levels of WT mice were unaffected following administration of either subcutaneous or intraventricular oxytocin (Fig. 7c, d). By contrast, the elevated distances travelled and average movement speeds of *Ager*^−/−^ mice in light-dark transition tests were normalised after intraventricular but not subcutaneous administration of oxytocin. Concordantly, intraventricular but not nasal administration of oxytocin normalised distances travelled by *Ager*^−/−^ mice in open field tests (Fig. 7e). These findings provide psychopharmacological support for the importance of vascular RAGE-dependent transport of peripheral oxytocin into the brain, which appears to be necessary for the development and maintenance of normal social behaviours.

## Discussion

Consistent with effects on maternal behaviour, mouse RAGE gene ablation (*Ager*^−/−^) mice leads to maternal neglect and dramatically decreased offspring survival. This phenotype is reminiscent of mice lacking oxytocin (*Oxt*^−/−^) or with deficiencies in oxytocin secretion (*Cd38*^−/−^) or action (*Oxtr*^−/−^)^5,38,39^. Notably, OT release from the hypothalamus was not affected by the RAGE-deficiency (Supplementary Figure 6). Supporting a primary role for oxytocin transport by endothelial RAGE, selective transgenic expression of RAGE in the endothelium of *Ager*^−/−^ mice rescued the maternal behaviour deficit. The potential for RAGE to serve as an oxytocin transporter is further supported by results from our biochemical binding studies, the in vitro BBB model system, where unidirectional blood to brain transport was seen, and by measurements of oxytocin in various regions of the CNS of WT and *Ager*^−/−^ male mice before and after peripheral administration of oxytocin. Oxytocin concentrations were consistently elevated in the third ventricle and cysterna as well as amygdala and PVN of WT but not *Ager*^−/−^ mice. c-Fos activation in the mPOA and BNST is a classical maternal behavioural response^8,51^, which was also recapitulated by exogenous oxytocin administration in WT but not *Ager*^−/−^ mice. These observations of peripheral oxytocin effectiveness indicate that hypothalamic oxytocin in *Ager*^−/−^ mice was not sufficient to induce maternal behaviour, suggesting the importance of central release of oxytocin stimulated by circulating oxytocin^59^.

The affinity of oxytocin binding to the oxytocin receptor has often been estimated to be *K*_D_ ~100 nM, although a high affinity form with *K*_D_ ~1 nM has also been reported^60^. Therefore our estimated *K*_D_ ~180 nM for binding between RAGE and oxytocin seems biologically reasonable. Under normal physiological conditions the primary effects of oxytocin are peripheral, and the endothelial RAGE transport system would not be engaged. However, during childbirth when both peripheral and central actions of oxytocin are needed, blood concentrations increase dramatically, which may saturate peripheral receptor occupancy and promote transport across the BBB. Levels of oxytocin in the periphery are lower than those in the CSF, in humans^16,61,62^, providing a concentration gradient for what appears to be a transport mechanism: this point of view depends on the methods of oxytocin measurements in blood, because a recent report has shown much higher blood OT levels than previously thought using a robust nanoLC-MS platform^63^. Endothelial RAGE signalling is not required for the central actions of oxytocin, much like the short form of the leptin receptor^64^. It remains unclear how oxytocin is transported by RAGE on the capillary endothelium. It is most likely that a vesicular trafficking system, involving endocytosis and transcytosis, a transcellular transportation across endothelial cells, is involved in oxytocin transportation mediated by RAGE carrier proteins^65^ (Supplementary Figure 4d).

Anterograde transport of oxytocin produced in the PVN and SON of the hypothalamus leads to its accumulation in axon terminals of the posterior pituitary, where it is released into the systemic circulation. Surges in the release and circulating concentrations of oxytocin occurring during childbirth activate oxytocin receptors in the reproductive organs^1,4^, including the uterus where oxytocin stimulates cervical dilation and uterine contractions and the mammary glands to stimulate lactation. Peripheral oxytocin may also act centrally to initiate maternal bonding behaviours^5,8^. Pharmacological studies clearly show that exogenous oxytocin has behavioural effects^5,8–12,24,66^, which further demonstrates that peripheral oxytocin can act centrally^17,18^. However, this has been a point of much debate and dissension^13,15,55,67^, as polypeptides typically do not cross the BBB without specific transport mechanisms^20,45^, and mechanisms for oxytocin transport were unknown. Findings presented here show that membrane-associated RAGE on endothelial cells and potentially elsewhere in the CNS binds to and transports peripheral oxytocin into the brain, as recently shown^16–19,66^.

It has been reported that intranasally administered oxytocin may be transported to the olfactory nerve via olfactory sensory neurons located in the mucous layer, or may reach the trigeminal nerve via trigeminal ganglion cell fibres, which are also located close to the surface of the nasal cavity^67,68^. Unfortunately, current experiments do not provide any evidence for such transport mechanisms. However, capillaries are dense in the mucus membrane, and thus the oxytocin can easily enter into the blood stream^17,68,69^. Once nasally applied oxytocin is conveyed by the blood stream, RAGE in the cerebral micro-vessels will transport oxytocin into the brain. RAGE transport seems to be responsible for the brain uptake of oxytocin nasally delivered oxytocin as treatment for human ASD patients or administration of megadoses of pitocin during labour, which can potentially affect maternal and foetal/newborn behaviour. Instead of direct nasal transport bypassing the BBB, one of the main mechanism may be uptake from the blood via RAGE, because rapid increases in plasma oxytocin concentrations have been reported^16;^ on the other hand, one study reported only a small increase in men^70^.

It has been reported that approximately 0.002% of the peripherally applied amount of oxytocin (5 μg) reaches the CNS at 10 min after subcutaneous or intraperitoneal administration in rats^71^, while the bioavailability is approximately 2% in rats, as measured by LC/MS after administration of 500 μg oxytocin in rats^72^. Rault et al. reported a bioavailability of 0.001% of i.n. application of 50 μg oxytocin in female pigs^18^. With an intracarotid artery bolus injection of ^125^I-labelled or ^3^H-labelled oxytocin, it was estimated that 1–2% of the oxytocin peptide accumulated on the BBB in rats^20^. In our study, the estimated availability of oxytocin in the CNF was 0.2%, after subcutaneous administration of 30 ng oxytocin in mice, which was a much lower amount of oxytocin than used in previous studies. The findings on oxytocin reaching the CNS were inconsistent among studies; this may be due to differences in treatment protocols, species, and detection methods. Further studies are required to determine the exact bioavailability.

The neural circuitry responsible for the peripartum acquisition of maternal bonding behaviours in rodents and mammals more generally is increasingly understood. Classical mapping studies revealed roles for the mPOA and adjacent vBNST^51–53,73,74^. Lesions in the mPOA identified important roles for dopaminergic neurons projecting to the ventral tegmental area (VTA) involved in reward and reinforcement learning^51,53^. Serotonergic signalling is also critical for the acquisition of maternal behaviour, as demonstrated by *Pet1*^−/−^ mice with diminished serotonin synthesis in the CNS^75^. The loss of maternal bonding by *Pet1*^−/−^ mice has been attributed to deficits in the mPOA and BNST, which are innervated by serotonergic neurons. oxytocin receptors expressed in the mPOA and BNST are also critical for normal maternal behaviour, which raises important questions about sites of oxytocin expression and mechanisms of oxytocin supply. Our findings suggest that peripheral oxytocin is transported by RAGE into the CNS to activate oxytocin receptors directly.

Finally, there is precedence for RAGE to serve as a peptide transporter across the BBB. For example, RAGE has been shown to transport amyloid β peptide across the BBB, which promotes Alzheimer disease-like symptoms in susceptible mice. RAGE has also been implicated in the pathogenesis of diabetes complications. Since both of these are deleterious, pathological effects, RAGE is being targeted for inhibition in drug discovery efforts^35^. By contrast, our findings demonstrate that RAGE is required for normal physiological functioning of oxytocin, which may also be inhibited by these drug discovery strategies. Parental bonding and nurturing and numerous other socially interactive behaviours are promoted by oxytocin and may either be missing in its absence or replaced by aggressive or anti-social behaviours.

Mass spectrometry of the eluted material from the immobilised esRAGE in human serum did not identify arginine-vasopressin; additionally, RAGE did not transport it in an in vitro blood–brain barrier (BBB) assay system (Supplementary Figure 7). In conclusion, the contribution of peripheral oxytocin to centrally-mediated behavioural actions has long been predicted^76^, and may now be understood and studied more precisely in view of the RAGE-mediated transport mechanism reported here. This transport mechanism is not gender-specific, but equally function in male and female mice (Fig. 4g).

## Methods

### Chemicals

Oxytocin was obtained from the Peptide Institute (Osaka, Japan). Liquid chromatography mass spectrometer (LC-MS)-grade water, acetonitrile (ACN), formic acid, trifluroacetic acid (TFA), and trichloroacetic acid (TCA) were purchased from Wako Chemicals (Tokyo, Japan). Stably-labelled oxytocin [^13^C,^15^N]oxytocin was synthesised using [^13^C_5_,^15^N_1_]-Pro_7_ and [^13^C_6_,^15^N_1_]-Leu_8_ (Scrum Co. Ltd., Tokyo, Japan), as described previously^73^.

### Animals

RAGE knockout (*Ager*^*−/−*^, deletion of the mouse gene of the receptor for AGE, *Ager*)^41^ and wild type (WT, RAGE^+/+^) mice (C57BL/6J) were produced by crossbreeding heterozygous mutant mice^27^. Endothelial RAGE-transgenic (Tg) mice^26^ were crossbred with *Ager*^*−/−*^ mice to yield endothelial RAGE-Tg *Ager*^*−/−*^ mice (KO + Tg). Both male and female mice were maintained under standard cage conditions (24 °C; 12-h light/dark cycle, lights on at 8:45 a.m.) with sawdust bedding, and food and water ad libitum. Breeding pairs were maintained in separate cages. Offspring were weaned at 21 days of age and housed in same-sex sibling pairs. For monitoring offspring survival, pregnant WT and *Ager*^*−/−*^ mice were transferred to a new environment (clean cages) 1 day before delivery. The offspring were either kept with biological mothers or caged with postpartum mothers of the alternative genotype. All animal experiments were approved by the Committee on Animal Experimentation of Kanazawa University and performed in accordance with the Fundamental Guidelines for Proper Conduct of Animal Experiment and Related Activities in Academic Research Institutions under the jurisdiction of the Ministry of Education, Culture, Sports, Science and Technology of Japan.

### Chromatography

Polypeptides were separated using a Superdex 75 pg HiLoad 26/600 column (GE Healthcare Japan, Tokyo) eluted at 1.0 ml/min with 5 mM ammonium acetate pH 7.8. Human serum (12 ml) preincubated with 48 ng oxytocin was separated and an enzyme immunosorbent assay (EIA) was used to identify OT in eluting fractions. Anti-RAGE monoclonal antibody (1.0 mg) or purified esRAGE (0.5 mg) were coupled to NHS-activated HiTrap (GE Healthcare). Sera samples (50 ml) from healthy consenting adults were applied to the HiTrap-anti-RAGE column equilibrated with 50 mM Tris–HCl (pH 7.4) and 0.15 M NaCl. Alternatively, the low molecular weight (<3000, Amicon Ultracel3K) fraction of human serum (50 ml) was applied to the HiTrap-esRAGE column previously equilibrated with phosphate-buffered saline (PBS). After washing the bound proteins were eluted with 100 mM glycine-HCl buffer (pH 2.5) for HiTrap-anti-RAGE or 10 mM Tris–HCl (pH 7.4) and 2.0 M NaCl for HiTrap-esRAGE and the eluates were analysed by EIA or LC-MS/MS and Western blotting.

### Surface plasmon resonance assay

Isolated human esRAGE was immobilised to BIAcore CM5 research grade sensor chips with the amine coupling kit to a density of ~5000 response units (RU)^27,32,41^. Oxytocin binding kinetics to the immobilised esRAGE was analysed using a BIAcore 2000 system (GE Healthcare Japan)^27,32,41^ and mobile phase 10 mM HEPES (pH 7.4), 0.15 M NaCl, 3 mM EDTA and 0.005% (V/V) surfactant P-20 at 25 °C and 20 μl/min. The sensor chips were regenerated with 10 mM NaOH and 0.5% SDS (W/V).

### Plate binding and competition assay

Oxytocin binding to RAGE and competition by RAGE ligands were assayed with oxytocin-coated 96-well plates, human esRAGE, and horseradish peroxidase (HRP)-conjugated anti-human RAGE antibody^27^. Oxytocin (100 μl, indicated concentrations) was immobilised in 96-well microtitre plates, 1 μg/ml esRAGE was added, and bound esRAGE was detected immunochemically using covalently coupled 〈RAGE antibody- horseradish peroxidise-catalysed oxidation of tetramethylbenzidine. RAGE ligands were S100B (Sigma-Aldrich, St. Louis, MO, USA)^30^, AGE-BSA (glyceraldehyde-derived AGE-BSA)^27,32,41^, amyloid β 1-42 (Sigma-Aldrich), and high-mobility group box 1 (HMGB1, Sigma-Aldrich)^42^.

### Luciferase assay

C6 rat glioma cells expressing RAGE and stably transformed with NF-κB promoter-driven luciferase constructs^27,41^ were incubated for 24 h in Dulbecco’s modified eagle’s medium (DMEM) supplemented with 0.1% foetal bovine serum (FBS) prior to 4 h stimulation with OT, S100B or AGE-BSA (glyceraldehyde-derived AGE-BSA)^32,41^. The luciferase activities were assayed with a Luciferase Assay System (Promega, Madison, MI, USA).

### Mass spectrometry

High MW proteins in diluted mouse plasma were precipitated with 2% TCA; supernatants obtained after centrifugation were analysed by LC-MS/MS. Mouse CSF samples diluted 1:5 with PBS (25 μl) were analysed directly. Human plasma samples (50 ml) spiked with 100 ng of oxytocin and 75 ng of [^13^C,^15^N]oxytocin were concentrated using immobilised esRAGE affinity chromatography. The eluting 1.0 ml fractions (Fig. 1h) were analysed by LC-MS/MS. The 25 μl samples were separated using a ZORBAX 300SB-C8 column (2.1 × 150 mm, 5 μm; CA, USA) eluted with a linear gradient of 0.1% (v/v) formic acid and acetonitrile (0–30%) at 0.3 ml/min and 50 °C. Eluted proteins were detected in the multiple reaction monitoring (MRM) mode of the MS/MS system (Shimazu UFLC, Kyoto, Japan; AB Sciex 4000 QTRAP, Framingham, MA, USA; positive ion electrospray (ESI) mode (capillary voltage 5 kV); desolvation voltage (75 V); curtain gas (nitrogen, 12 l/h, 65 psi and 600 °C); nitrogen gas pressure (65 psi), and collision voltage (30 V). Oxytocin MRM transition ions and fragment types were *m*/*z*: 1008.2 → 723.2 (b6); and [^13^C,^15^N]oxytocin: *m*/*z*: 1021.2 → 723.2 (b6)^73^. Data were acquired and processed with AB Sciex Analyst Version 1.4.1.

### Blood-brain barrier (BBB) model

For the in vitro BBB model (MBT-24H, PharmaCo-Cell Co., Nagasaki, Japan), rat brain pericytes (1.5 × 10^4^ cells/cm^2^) and rat astrocytes (1.5 × 10^4^ cells/cm^2^) were seeded on the bottom of the collagen-coated polyester membranes of Transwell inserts (Corning Life Sciences, MA, USA). After 16 h the cells firmly adherent, monkey brain vascular endothelial cells (1.5 × 10^5^ cells/cm^2^) were then seeded on the upper side of the inserts in 24-well culture plates. The in vitro BBB models were established within 4 days of seeding the cells according to the manufacturer’s instructions^46^. Trans-endothelial electrical resistance (TEER), which primarily reflects the flux of sodium ions through the cell layers in the culture conditions, was measured with an epithelial-volt-ohm metre and an Endohm-6 chamber electrodes (World Precision Instruments, Sarasota, FL, USA). The TEERs of the coated, cell-free filters were subtracted from the measured TEER values of the models and shown as Ω cm^2^. The apparent permeability constants (Papp) between luminal and abluminal chambers were calculated from the distribution ratios^46^. Endothelial expression was silenced using RAGE shRNA and the pSilencer 3.0-H1 vector (Ambion, Austin, TX, USA)^41^.

### Flow cytometry

Isolated brain endothelial cells were washed and resuspended in staining buffer (PBS containing 2% FCS) containing FcBlock (BD Biosciences, San Jose, CA, USA). The cells were incubated with polyclonal rabbit anti-RAGE antibody or isotype IgG (15 min at 4 °C in the dark)^27^ followed by anti-rabbit IgG-FITC (eBioscience, San Diego, CA, UAS), and analysed by FACS AriaII (BD Biosciences) and FlowJo software (Tree Star, Inc., Ashland, OR, USA).

### Immunohistochemical analysis

The hippocampus and choroid plexus were cut into 10-μm-thick sections by using a cryostat. The sections were blocked in PBS containing 0.3% TritonX-100 and 3% BSA for 1 h at room temperature, and incubated at 4 °C overnight with primary polyclonal rabbit anti-RAGE antibody (1:1000), anti-CD31 or anti-Caveolin 1 antibodies (Millipore, Billerica, MA, USA; 1:500) and 4′,6-diamidino-2-phenylindole (DAPI, Dojindo; Kumamoto, Japan, 1:2000) as previously described^20^. The sections were subsequently washed with 0.3% TritonX-100 in PBS and incubated with Alexa Fluor 488 (1:200, Invitrogen Molecular Probes) and Cy3-labled-IgG (1:100, Jackson ImmunoResearch Laboratories, PA) for 1 h at room temperature. Imaging was performed with a Nikon EZ-C1 laser confocal microscope (Tokyo, Japan).

### Sampling of the cerebrospinal fluid (CSF) and blood

CSF was collected according to the protocol for sampling CSF from mice without detectable plasma contamination^77,78^. Under ketamine anaesthesia (100 mg/kg intraperitoneally), the occipital skull was exposed, and the dura mater of the cisterna magna appeared as a glistening and clear reverse triangle through which the medulla oblongata, a major blood vessel (arteria dorsalis spinalis), and CSF space were visible. A capillary tube was inserted into the cisterna magna through the dura mater, blood-free CSF samples were collected with a 32G needle and immediately frozen with dry ice prior to storage at −80 °C. After CSF collections, blood samples were obtained by cardiac puncture from the mice under continuous ketamine anaesthesia.

### Microperfusion

For microperfusion probe implantation^79^, the male mice were with a subcutaneous injections of ketamine. The heads of anesthetised mice were fixed in a stereotactic frame (Narishige, Tokyo, Japan) and a 1 mm hole was drilled in the skull. The intact dura intact was then punctured to create defined opening of the meninges. Using the stereotactic frame, a healing dummy was slowly inserted into the three brain positions^80^. The probe was fixed to the skull by using two anchor screws and biocompatible dental cement. All surgical procedures were completed within 30 min. A healing dummy was used to provide mechanical stability during the implantation and the healing period of 2 weeks^80^. The microperfusion probe (a 4-mm length of the coaxial tube, 2.5 mm in diameter) consisting of a 20 G fluorinated ethylene propylene guide cannula was replaced before sampling with an inflow/outflow tubing on the day of the experiments^80^. This tubing was connected to two glass Hamilton microsyringes placed in syringe pumps (Eicom, Osaka, Japan). Microperfusate was pumped into the probe at a flow rate of 2 µl/min, and the samples were withdrawn at the same flow rate. Sampling was conducted for 2 h. Both microprobes were perfused without sampling for 60 min before the first 30-min microperfusates from the amygdala, PVN, and prefrontal cortex were collected. The microperfusates were mixed under sterile conditions and consisted of 154 mM NaCl, 2.2 mM CaCl_2_, 5.6 mM KCl, 2.3 mM NaHCO_3_, and 0.15% BSA (pH 7.4)^80^. Beginning immediately after the nasal application, 4 additional microperfusates were taken at 30-min intervals. After the termination of the experiments, the brains were removed and snap-frozen to obtain 40-μm cryo-cut stained brain slices later for the histological verification of the perfusion site.

### Administration of oxytocin

Synthetic oxytocin (1000 ng/ml × 20 μl) was administered nasally; the solution was applied bilaterally on the rhinarium, which is the area referred to as the glaberous skin around the nostrils, using a pipette), intravenously (100 ng/ml × 30 μl) through the tail vein, or subcutaneously (100 ng/ml × 0.3 ml) into the shoulder skin. [^13^C,^15^N]oxytocin (100 μg/ml × 0.3 ml) was subcutaneously injected.

### Enzyme immunoassay for oxytocin

Oxytocin immunoreactivity levels in serum and CSF were quantified by using an oxytocin EIA kit (Enzo Life Sciences, NY, USA, formerly Assays Designs, MI, USA), following the manufacturer’s manual. The CSF samples (5 μl) were thawed and diluted 1:20 in assay buffer^77^. CSF samples were assayed without protein extraction due to the low protein concentration in the CSF. The plasma samples (100 μl) were thawed on ice and assayed with protein extraction^5,77^, whereas an additional extraction step may have resulted in lower than actual oxytocin levels. Each sample volume was so small, the CSF was assayed without extraction, though unextracted samples result in binding of complexes of oxytocin stuck to interacting proteins. The oxytocin assay had two linear ranges, which covers a lower concentration range from a few to 30 pg/ml, and a higher concentration range between 50 and 1000 pg/ml. The inter-assay and intra-assay coefficients of variation were less than 15%.

### Oxytocin release from the hypothalamus

WT and *Ager*^*−/−*^ male mice were anaesthetised with pentobarbital sodium (50 mg/kg). The entire bilateral hypothalami were obtained and placed separately in different wells of a 12-well plate with 0.4 ml normal Locke’s solution (pH 7.25) containing 140 mM NaCl, 5 mM KCl, 1.2 mM MgCl_2_, 2.2 mM CaCl_2_, 10 mM glucose, 10 mM HEPES, and 0.01% BSA, in a 35 °C water bath. The incubation medium was replaced 10 times every 3 min, as described^80^. Beginning at the 11th replacement, the temperature was shifted to 38.5 °C. From the 13th–15th replacements, cyclic ADP-ribose (a final concentration of 100 μM, Sigma) was added into the Locke’s medium at 38.5 °C. Aliquots from the 8th replacement were preserved at −80 °C for assaying the oxytocin concentrations released from the hypothalamus.

### Light-dark transition test

The light-dark transition was used to examine the anxiolytic-like or anxiogenic-like activity of the mice as previously described^81^. The light-dark test chamber (200 mm × 600 mm × 200 mm) consisted of two dividing rooms: a small dark (2 lux) safe compartment (one-third, 200 mm × 200 mm × 200 mm—dark box) and a large brightly illuminated (250 lux) aversive compartment (two-thirds, 400 mm × 200 mm × 200 mm—light box). The mice were placed into the light arena and were allowed to move freely between the two chambers for 600 s. Each male mouse was placed in the centre of the light chamber, and the mouse was allowed to run freely between the two chambers for 10 min. The trial was recorded for 10 min by using the ANY-maze video system (Stoelting Co., Wood Dale, IL, USA). Latency to enter (defined by all four paws entering), time spent, entries, and distances travelled in the light chamber were recorded. A recovery test was performed following subcutaneous injection of oxytocin (100 ng/ml × 0.3 ml) or intraventricular application of oxytocin (100 ng/ml) for 10 min at 2 μl/min, through implanted cannulae, as described above.

### Open field test

The open field test is meant to assess anxious behaviour^80^. It uses a wooden box (60 × 60 × 20 cm) covered with polypropylene and having an outlined centre space (30 × 30 cm). Animal are in the box for 10 min, and their movements into the centre are digitally recorded and analysed using ANY-maze software. This test is based on the idea that mice naturally prefer to be near protective walls rather than being exposed to potential dangers in open spaces. Test chambers are cleaned after each trial.

### Cerebral ischaemia model

WT and *Ager*^*−/−*^ male mice (8 to 12-weeks-old and weighing 20–30 g) were used for the experiments. The animals were anaesthetised with 2.0% halothane and maintained with 0.5% halothane through a facemask. Brain ischaemia was induced via bilateral occlusion of the common carotid arteries (BCCAO) for 15 min using microvascular clips as described previously^23^. Laser-Doppler flowmetry was used to measure cerebral cortical microperfusion (3 mm lateral to bregma). In our experimental model, the mice that exhibited <15% of the baseline control microperfusion during the first minute of occlusion were used in subsequent experiments. Rectal temperature was maintained at 36.5–37.5 °C by using a heat lamp and a blanket until the mice were completely alert. The control animals underwent a sham-operation that was identical with the exception of the occlusion. To quantify accumulative BBB leakages, 200 µl of sodium fluorescein (Sigma-Aldrich) at a concentration of 6 mg/ml in PBS was injected via the venous sinus of retro-orbital in the mice. Sodium fluorescein (MW 376.3) is a fluorescent tracer that does not cross an intact BBB. Sixty minutes later, the CSF and blood were collected according to the protocol for sampling of CSF and blood from mice^80^. The fluorescence of CSF and serum were measured at 460 nm at an excitation wavelength of 355 nm by using a Fluoroscan Ascent FL luminometer (Labsystems. Wilmington, DE, USA). Results are presented as relative fluorescence units (CSF value/serum value) (%).

### c-Fos activity

The c-Fos immunohistochemistry was performed as previously described^53^. Briefly, anesthetized male mice were intracardially perfused with cold PBS followed by a cold 4% paraformaldehyde (PFA) in PBS. The brains were removed and post-fixed in a 4% PFA solution overnight at 4 °C. Brain regions were cut into 2–4 large blocks. The blocks were sliced on a microtome into 20-μm-thick sections. The sections were pre-incubated in blocking solution (3% bovine serum albumin and 0.3% Triton X-100 in PBS) for 1 h, then incubated with an anti-c-Fos antibody (sc-52, 1:200; Santa Cruz Biotechnology, Santa Cruz, CA, USA) in the blocking solution for 12 h at 4 °C. After three washes with washing buffer, the sections were incubated with goat anti-rabbit IgG antibody coupled with Alexa Fluor 488 (Invitrogen, Carlsbad, CA, USA) in the blocking solution for 1 h at room temperature. The images were obtained by using an Olympus IX71 inverted microscope equipped with a cooled CCD camera (Cool SNAP HQ2; Roper Scientific, Tucson, AZ, USA). The number of c-Fos immuno-positive nuclei in each brain section were recorded and analysed using Metamorph software (Molecular Devices, Downingtown, PA, USA).

### Immunoelectron microscopy

The immunogold staining method was applied to the amygdala as previously described^5^. Briefly, after 10 min of oxytocin (100 ng/ml × 0.3 ml) injection, wild type (WT, *Ager*^+/+^) and *Ager*^*−/−*^ mice were perfused with a mixture containing 2% paraformaldehyde and 2% glutaraldehyde solution in 0.1 M phosphate buffer (pH 7.2). The tissue blocks were then fixed by immersion for 4 h at 4 °C in the same solution and washed for 1 h with 0.1 M phosphate buffer (pH 7.2). After washing, the tissue blocks were dehydrated and embedded in LR-White resin (London Resin Co.). Ultrathin sections were mounted on nickel grids. The sections were washed with PBS, incubated in a blocking solution of 1% bovine serum albumin (BSA) and 0.05% sodium azide (NaN_3_) in PBS for 15 min, and then exposed with anti-oxytocin polyclonal antibody (1:5000, Chemicon International, Inc. USA) overnight at 4 °C. After washing twice with PBS, the sections were incubated with 5 nm gold-conjugated goat anti-rabbit secondary antibody (1:100, Sigma, USA) in a solution containing 0.1% BSA in PBS for 4 h at room temperature. The sections were washed with PBS and then with distilled water. After washing, the sections were stained with uranyl acetate and analysed under a transmission electron microscope (Joel, Tokyo Japan) by using an 80-kV accelerating voltage.

### Statistical analysis

*P* values were calculated by using two-tailed Student’s *t*-test for pair wise comparisons, and one-way or two-way analysis of variance (ANOVA) followed by Bonferroni’s or Tukey’s test for multiple comparisons, unless otherwise stated. The Kaplan–Meier survival analysis was performed to compare survival curves between the different groups of mice. A *P-*value of < 0.05 was considered statistically significant. Data are expressed as mean ± SEM. Statistical data with F vales are shown in Supplementary Table 1.

### Reporting summary

Further information on experimental design is available in the Nature Research Reporting Summary linked to this article.

## Supplementary information

Description of Additional Supplementary Files

Supplementary Information

Supplementary Data 1

Reporting Summary

## Data Availability

The data that support the findings in this study are available from the corresponding author upon reasonable request. The source data of each figure are presented as a Supplementary Data 1.
