# Supplementary material for: Vascular RAGE transports oxytocin into the brain to elicit its maternal bonding behaviour in mice
Source: Commun Biol. 2019 Feb 25;2:76. doi: 10.1038/s42003-019-0325-6 (PMC6389896; doi:10.1038/s42003-019-0325-6)
Supplement: Supplementary file 1 — Description of Additional Supplementary Files [file 42003_2019_325_MOESM1_ESM.docx]

**Description of Additional Supplementary File**

**File Name**: Supplementary Data 1

**Description**: Supplementary Tables (excel file). Source data of the main Figures.

**File Name**: Supplementary Information

**Description**: Supplementary Figures (pdf file). Supplementary Figures 1-7. Supplementary Table 1 (pdf file). The statistical analysis results.
