# Supplementary material for: Vascular RAGE transports oxytocin into the brain to elicit its maternal bonding behaviour in mice
Source: Commun Biol. 2019 Feb 25;2:76. doi: 10.1038/s42003-019-0325-6 (PMC6389896; doi:10.1038/s42003-019-0325-6)
Supplement: Supplementary file 2 — Supplementary Information [file 42003_2019_325_MOESM2_ESM.pdf]

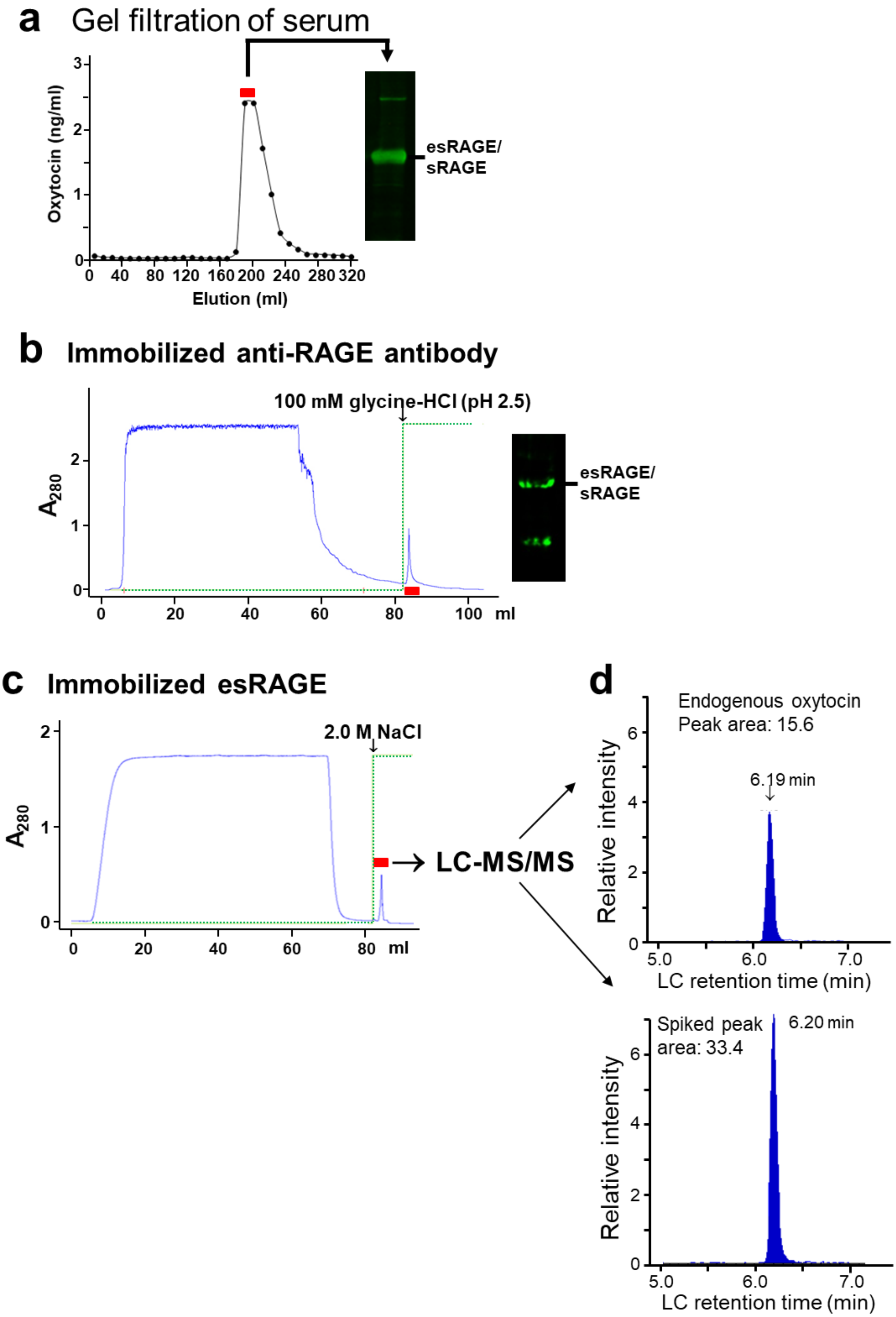

Supplementary Figure 1. Oxytocin-RAGE complexes in human sera. (**a**) A peak fraction of oxytocin from human sera contained sRAGE/esRAGE after separation by gel permeation chromatography (Superdex 75pg HiLoad 26/600 column eluted with 5 mM ammonium acetate buffer, pH 7.8). (**b**) Anti-RAGE affinity chromatography with human serum (50 ml). Bound proteins were eluted with 100 mM glycine-HCl buffer (pH 2.5) and fractions were analysed by oxytocin EIA and Western blotting with anti-RAGE antibody. (**c**) Human esRAGE affinity chromatography of human serum (50 ml) spiked or not with synthetic oxytocin (100 ng). (**d**) Protein eluted with 1 M NaCl was analysed by oxytocin EIA and a liquid chromatography-tandem mass spectrometry (LC-MS/MS) results for endogenous oxytocin (left) and same serum spiked with 100 ng oxytocin (right).

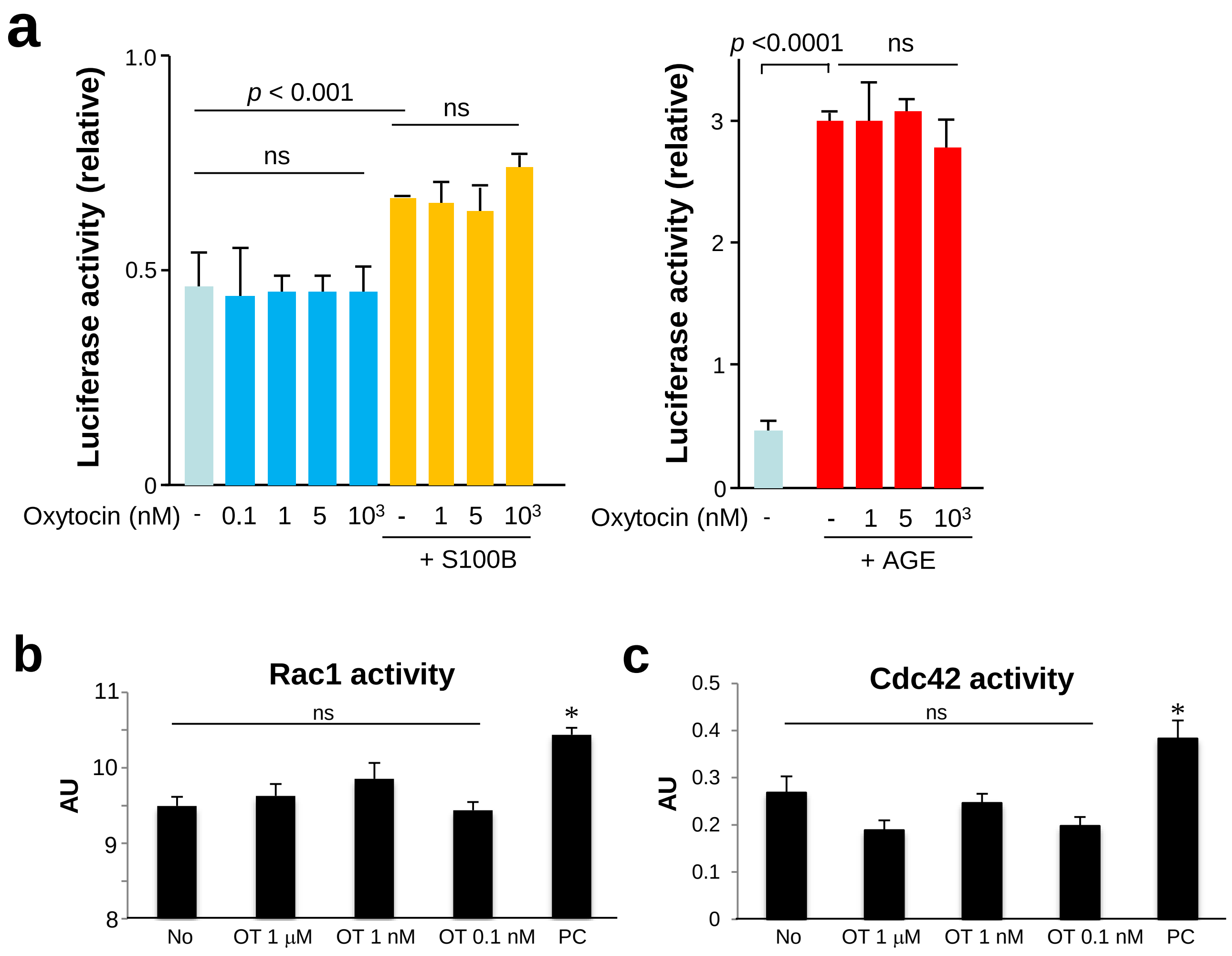

Supplementary Figure 2. RAGE signalling assay. (**a**) Indicated concentrations of oxytocin were added to culture media with or without S100B (50 μg/ml) or AGE-BSA (AGE, 50 μg/ml) of human mRAGE-expressing luciferase reporter gene-carrying rat C6 glioma cells (n = 3). Indicated concentrations of oxytocin were added to the culture media of human mRAGE-expressing rat C6 glioma cells. Rac1 (**b**) or Cdc42 (**c**) activities were then measured by G-LISA Rac1 and Cdc42 activation assay biochem kits (Cytoskelton), respectively (n = 3). ns, not significant; PC, positive control; OT, oxytocin. All graphs show mean ± SEM. *$P < 0.05$.

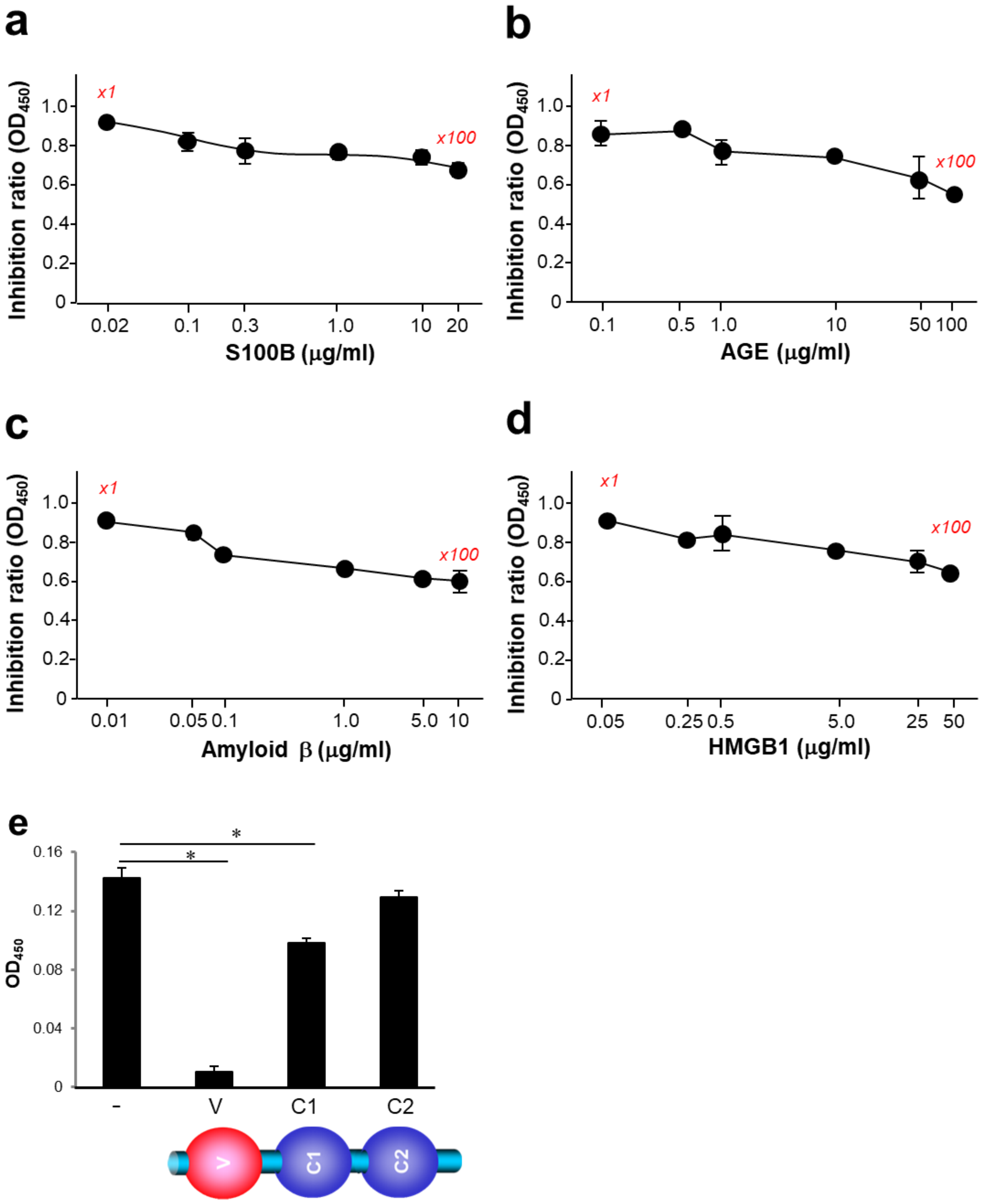

Supplementary Figure 3. (a–d) Plate competition assays. Preincubation mixtures of esRAGE (1 µg/ml) with indicated concentrations of S100B (**a**), AGE-BSA (AGE) (**b**), amyloid β (**c**), or high-mobility group box 1 (HMGB1) (**d**) were added into wells for 30 min on which oxytocin (100 µM) had been immobilized. The formation of oxytocin–RAGE complexes was detected with horseradish peroxidase (HRP)-conjugated anti-RAGE antibody (n = 3). All graphs show mean ± SEM. (**e**) To examine oxytocin-binding site on RAGE, synthetic V, C1, and C2 domains (30 µg/ml) were used with this system (n = 3). The formation of oxytocin–RAGE complexes was detected with horseradish peroxidase (HRP)-conjugated anti-RAGE antibody (n = 3). *$P < 0.05$. All graphs show mean ± SEM.

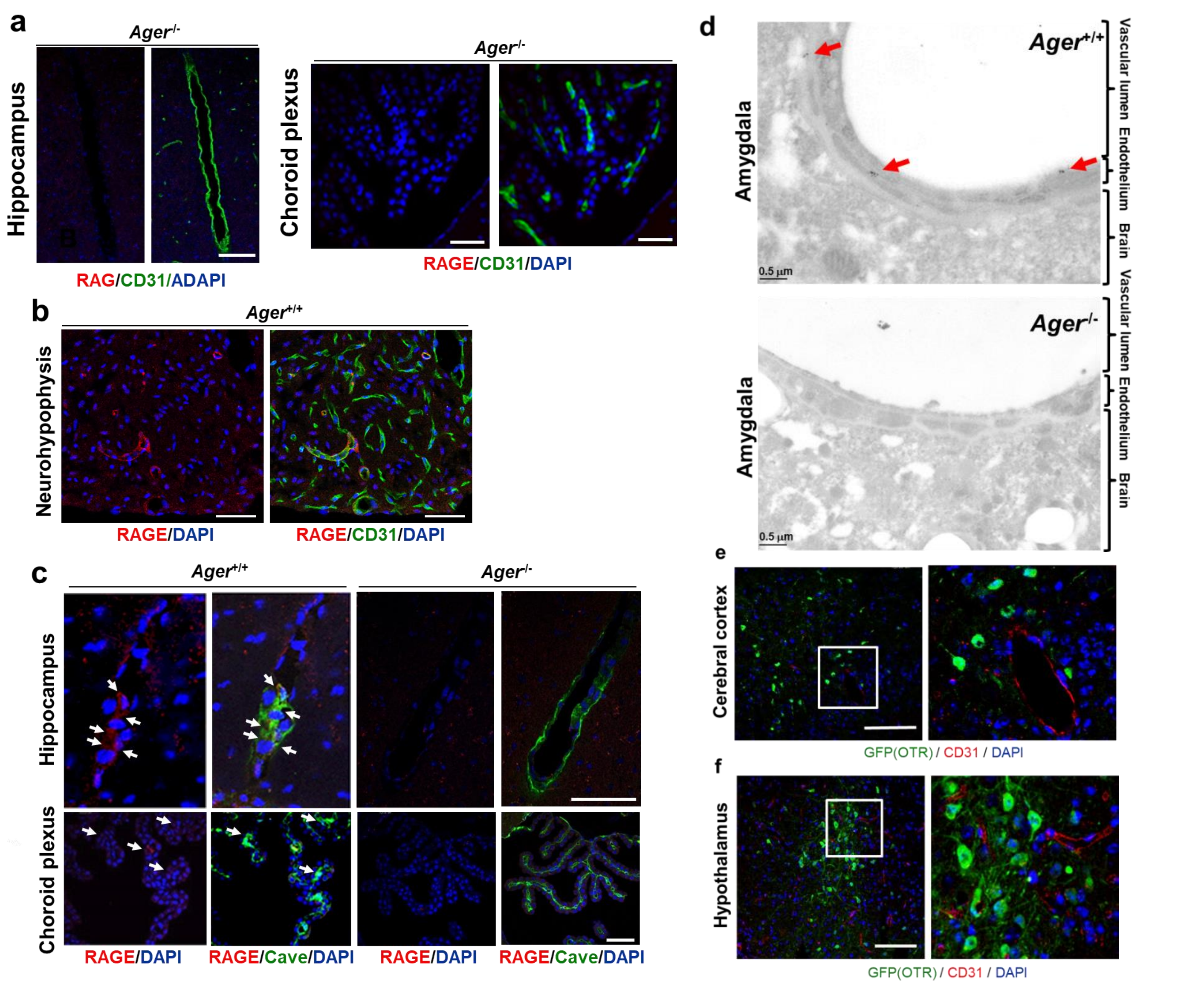

Supplementary Figure 4. Expression of RAGE in the brain vasculature and immunoelectron microscopical analysis for oxytocin detection. (**a**) Confocal microscopy. Sections of the (**a**, left) hippocampus (CA1, stratum radiatum) and (**a**, right) choroid plexus in the third ventricle of *Ager*$^{-/-}$ (RAGE$^{-/-}$) male mice were immunostained with anti-RAGE and anti-CD31 antibodies; nuclei were stained with DAPI (Bar = 100 μm). (**b**) RAGE expression was observed in endothelial cells of the neurohypophysis, one of the circumventricular organs, which are located along the midlines of the brain ventricles (Bar = 100 μm). (**c**) RAGE expression in the vascular cells in the hippocampus CA1 region (stratum radiatum) and the choroid plexus in the third ventricle. Confocal microscopic analyses by using wild type (WT, *Ager*$^{+/+}$) and *Ager*$^{-/-}$ mice. Brain sections were subjected to immunostaining with anti-RAGE and anti-caveolin 1 (Millipore, Billerica, MA, USA; 1:500) antibodies. The nuclei are stained with 4',6-diamidino-2-phenylindole (DAPI, green). Co-staining of RAGE (red) and caveolin 1 (Cave, green) is observed in the endothelial cells indicated by arrows. Bar, 100 μm. (**d**) Immunoelectron microscopy. Red arrows indicate immunogold signals of within endothelial cells and in subendothelial spaces of the amygdala from WT (*Ager*$^{+/+}$), but not from *Ager*$^{-/-}$ mice. The immunogold staining method was applied to the amygdala as previously described (5). Briefly, the amygdalae of the 2 genotypes of mice that had been injected with oxytocin (300 ng/kg) 10 min previously were perfused with a mixture containing 2% paraformaldehyde and 2% glutaraldehyde solution in 0.1 M phosphate buffer (pH 7.2). After perfusion, the tissue blocks were fixed by immersion for 4 h at 4 ºC in a same solution and then washed for 1 h with 0.1 M phosphate buffer (pH 7.2). After washing, the tissue blocks were dehydrated and embedded in LR-White resin (London Resin Co.). Ultrathin sections were mounted on nickel grids. The sections were washed in PBS and incubated in a blocking solution containing 1 % BSA and 0.05 % NaN_3 in PBS for 15 min. After washing in PBS, the sections were incubated with anti-oxytocin polyclonal antibody (1 : 5000, Chemicon International, Inc. USA) and diluted in PBS overnight at 4 ºC. After washing twice in PBS, the sections were incubated with 5 nm gold-conjugated goat anti-rabbit secondary antibody (1:100, Sigma, USA) and diluted in solution containing 0.1% BSA in PBS for 4 h at room temperature. The sections were washed in PBS and then washed in distilled water. After washing, the sections were stained with uranyl acetate and analysed in a transmission electron microscope (Joel, Tokyo Japan) using an 80-kV accelerating voltage. (**e** and **f**) Expression of oxytocin receptor (OTR) in the brain. Immunohistochemical detection of the OTR by using OTR-reporter mice (Venus mice). Coronal brain sections of the cerebral cortex (**e**) and hypothalamus (**f**) from the OTR-reporter mice were subjected to immunohistochemical analyses. Right panels are the enlarged images of the square parts in the left panels. Note that the OTR (Venus) is not co-localized with CD31-positive endothelial cells. Bars indicate 100 μm.

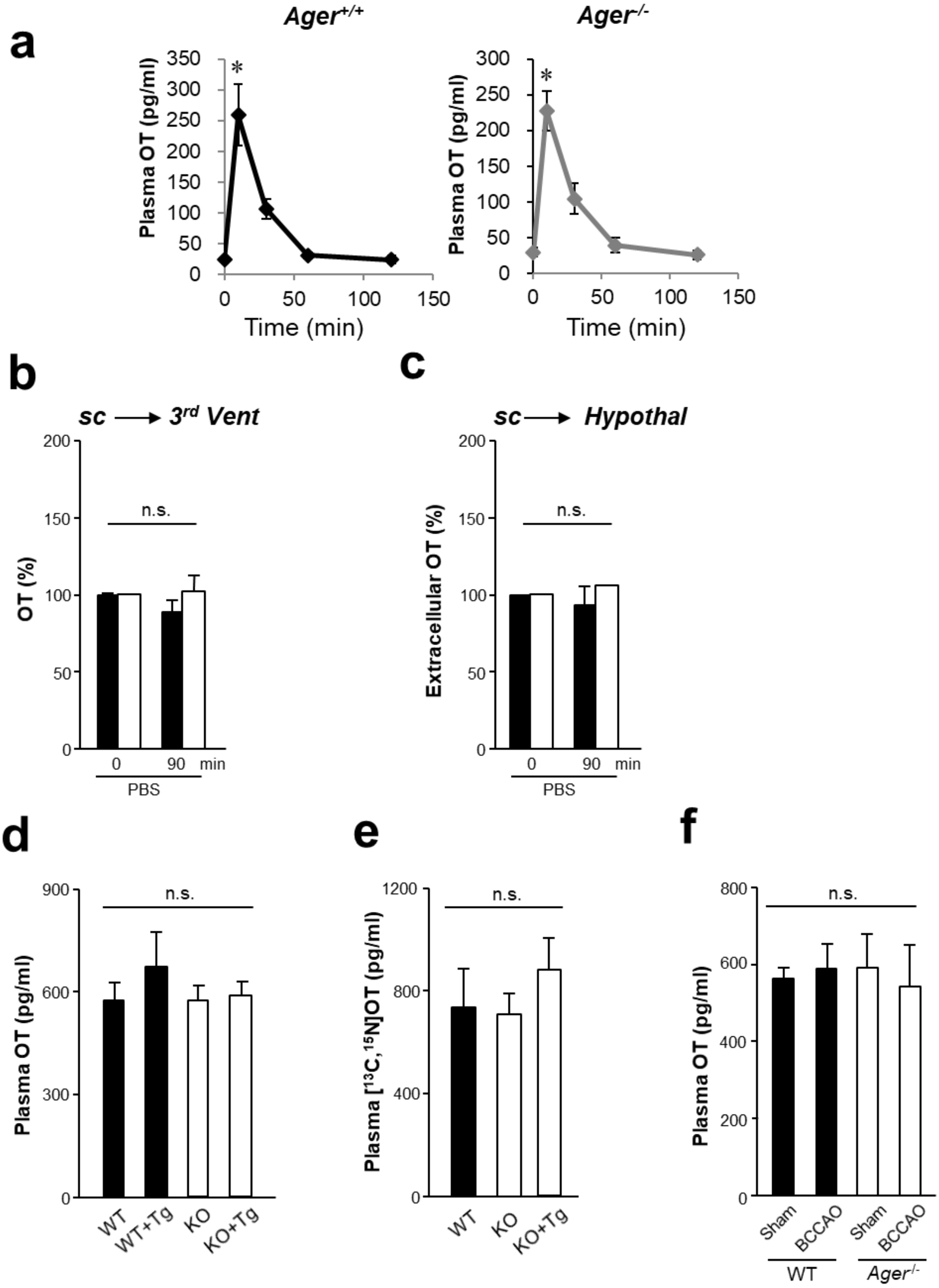

Supplementary Figure 5. Transportation of peripherally administrated oxytocin (OT) or PBS into the blood or brain. (**a**) Plasma oxytocin concentration kinetics after subcutaneous (sc) injection of oxytocin (100 ng/ml x 0.3 ml). Blood was recovered from the cardiac ventricles of the wild type (WT, $RAGE^{+/+}$) and *Ager*$^{-/-}$ ($RAGE^{-/-}$) male mice (n = 3–16). Oxytocin was measured in the extracted samples. *$P < 0.05$. (**b** and **c)** Oxytocin concentrations in the CSF of the third ventricles (**b**) and extracellular fluid of the hypothalami (PVN) (**c**) after 90 min of sc (0.3 ml) injections of PBS, respectively (n = 5). (**d** and **e**) Plasma oxytocin concentrations of the mice in Figures 3e, 4f and 5g. Blood was recovered from the cardiac ventricles of the wild type (WT, $RAGE^{+/+}$), *Ager*$^{-/-}$ (KO, $RAGE^{-/-}$) mice, and mice overexpressing human RAGE in endothelial cells in wild type (WT, $RAGE^{+/+}$) (WT + Tg) and *Ager*$^{-/-}$ ($RAGE^{-/-}$) (KO + Tg) mice (n = 5–14). Oxytocin concentrations in the plasma were measured 60 min after subcutaneous injections of oxytocin (**d**) or [^{13}C,^{15}N]oxytocin (**e**). (**f**) Mouse bilateral common carotid arteries occlusion (BCCAO) model and the sham operated control (Sham) were used. Oxytocin concentrations in the plasma were measured 60 min after subcutaneous injections of oxytocin or [^{13}C,^{15}N]oxytocin. Oxytocin concentrations were measured with enzyme immunosorbent assay (EIA) or LC-MS/MS. Values are mean ± SEM. n.s., not significant.

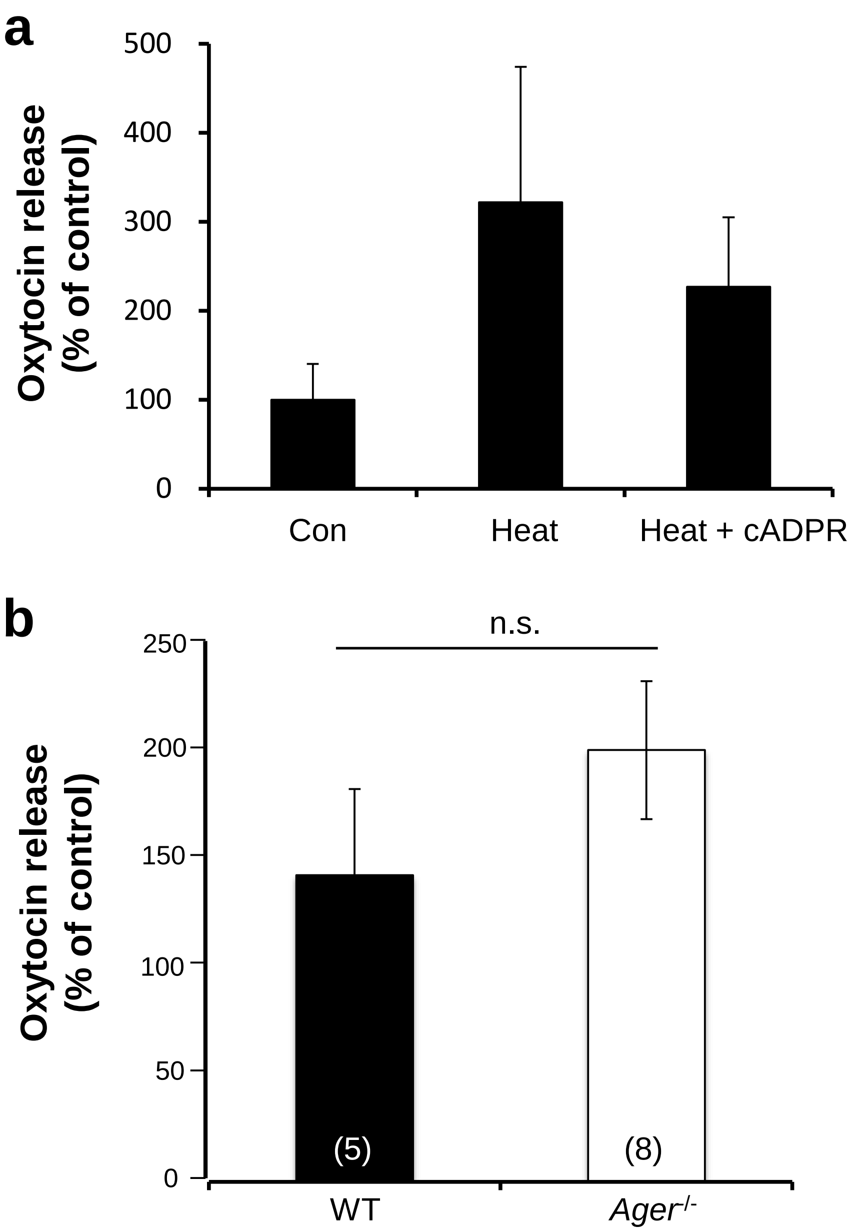

Supplementary Figure 6. Oxytocin release from the hypothalamus by simultaneous stimulation with cADP-ribose and heat in WT and *Ager$^{-/-}$* male mice. (**a** and **b**) Oxytocin release from the whole hypothalamus incubated with 100 μM cyclic ADP-ribose for 3 min, after the incubation temperature shift from 35˚C to 38.5˚C (68). Data are shown as ratio of change in oxytocin level to that of basal level (n = 4–8). Time interval is 3 min. All graphs show mean ± SEM. n.s., not significant.

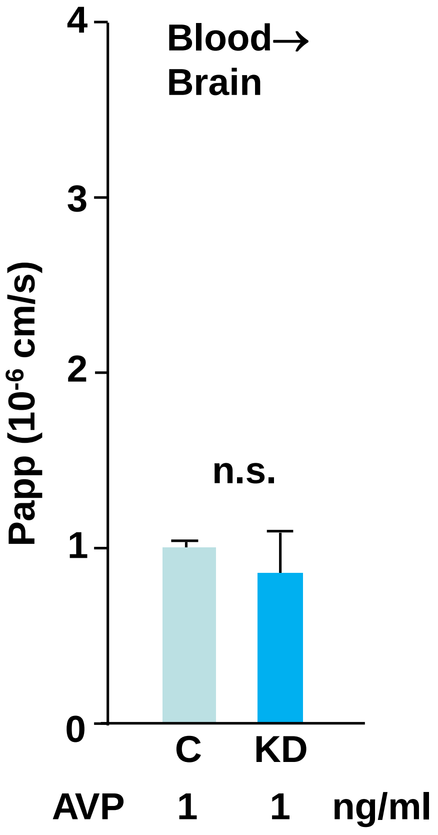

Supplementary Figure 7. RAGE do not transport arginine-vasopressin (AVP) across an in vitro blood brain barrier (BBB). Arginine-vasopressin (1.0 ng/ml) was added to the upper (luminal, blood) chambers of the model BBB system, and 3 h later arginine-vasopressin was quantified in the lower (abluminal, brain) chambers. The apparent permeability constants (Papp) for transfer were calculated from the distribution ratios across the chambers (n = 4). The endothelial cells used in the upper chamber were treated with RAGE shRNA (knockdown, KD) or control (C) vectors to assess the effects of RAGE knockdown. Arginine-vasopressin level was measured by an ELISA kit (ADI-900-017, Enzo Life Sciences, NY, USA). Values are mean ± SEM. n.s., not significant.

Supplementary Table 1. The statistical analysis results

			Method of follow-up test (post hoc test)
Fig. 1a	Kaplan-Meier survival analysis		
Fig. 1b	Kaplan-Meier survival analysis		
Fig. 2b	Two-way ANOVA for interaction	$F_{2,24}$ = 0.4539, P = 0.5069	Tukey’s
	Two-way ANOVA for treatment	$F_{1,24}$ = 7.262, P = 0.0127	
	Two-way ANOVA for C vs.KD	$F_{1,24}$ = 13.73, P = 0.0011	
Fig. 2e	Two-way ANOVA	$F_{2,24}$ = 12.25, P = 0.0018	Tukey’s
Fig. 2f	-Two-way ANOVA	$F_{2,24}$ = 29.14, P = 0.0001	Tukey’s
Fig. 4a	Two-way ANOVA for time	$F_{1,24}$ = 32.15, P = 0.0001	Bonferroni’s
Fig. 4b	One-way ANOVA	$F_{1,16}$ = 6.31, P = 0.0010	Bonferroni’s
Fig. 4b	-Two-way ANOVA for time	$F_{1,24}$ = 32.15, P = 0.0001	Bonferroni’s
Fig. 4c	One-way ANOVA for time	$F_{1,11}$ = 5.99, P = 0.0010	Bonferroni’s
Fig. 4d	One-way ANOVA for iv	$F_{1,4}$ = 16.99, P = 0.0125	Bonferroni’s
	One-way ANOVA for nasal	$F_{1,4}$ = 48.12, P = 0.0003	Bonferroni’s
	One-way ANOVA for sc	$F_{1,4}$ = 17.13, P = 0.0200	Bonferroni’s
	Two-way ANOVA for interaction of all	$F_{5,78}$ = 19.43, P = 0.0001	Bonferroni’s
Fig. 4e	Two-way ANOVA for genetype	$F_{1,57}$ = 4.090, P= 0.0478	
Fig. 4g	Two-way ANOVA for interaction	$F_{1,21}$ = 5.980, P = 0.0234	
	Two-way ANOVA for treatment	$F_{1,21}$ = 5.966, P = 0.0235	
Fig. 5d	Two-way ANOVA for interaction	$F_{2,16}$ = 7.970, P = 0.0025	Bonferroni’s
Fig. 6b	One-way ANOVA	$F_{15,64}$ = 11.24, P = 0.0001	Tukey’s
Fig. 7a	Kaplan-Meier survival analysis		
Fig. 7b	Kaplan-Meier survival analysis		
Fig. 7c	Student’ t-test		
Fig. 7d	Student’ t-test		
Fig. 7e	One-way ANOVA	$F_{2,21}$ = 34.28, P = 0.0001	Bonferroni’s

Suppl. Fig. 2a	One-way ANOVA	$F_{4,55}$ = 3.563, P = 0.0001	Bonferroni's
		$F_{4,35}$ = 36.42, P = 0.0001	Bonferroni's
Suppl. Fig. 2b	One-way ANOVA	$F_{4,35}$ =4.023, P = 0.0084	Bonferroni's
Suppl. Fig. 2c	One-way ANOVA	$F_{4,35}$ = 7.727, P = 0.0001	Bonferroni's
Suppl. Fig. 3a	One-way ANOVA	$F_{5,42}$ = 17.37, P = 0.0001	Bonferroni's
Suppl. Fig. 3b	One-way ANOVA	$F_{5,42}$ = 34.30, P = 0.0001	Bonferroni's
Suppl. Fig. 3c	One-way ANOVA	$F_{5,42}$ = 20.18, P = 0.0001	Bonferroni's
Suppl. Fig. 3d	One-way ANOVA	$F_{5,42}$ = 15.38, P = 0.0001	Bonferroni's
Suppl. Fig. 3e	One-way ANOVA	$F_{5,42}$ = 152.1, P = 0.0001	Tukey's
Suppl. Fig. 5a	One-way ANOVA	$F_{4.43}$ = 6.872, P = 0.0002	Bonferroni's
	One-way ANOVA	$F_{4.24}$ = 4.210, P = 0.0101	Bonferroni's
